# A call to advance and translate research into policy on governance, ethics, and conflicts of interest in public health: the GECI-PH network

**DOI:** 10.1186/s12992-021-00660-0

**Published:** 2021-01-25

**Authors:** Rima Nakkash, Melissa Mialon, Jihad Makhoul, Monika Arora, Rima Afifi, Abeer Al Halabi, Leslie London

**Affiliations:** 1 Faculty of Health Sciences, American University of Beirut, Beirut, Lebanon; 2 School of Public Health, University of São Paulo, São Paulo, Brazil; 3 Trinity Business School, Trinity College Dublin, Dublin, Ireland; 4 Public Health Foundation of India, New Delhi, India; 5 Department of Community and Behavioral Health, College of Public Health, University of Iowa, Iowa, USA; 6 School of Public Health and Family Medicine, University of Cape Town, Cape Town, South Africa

**Keywords:** Commercial determinants of health, Ethics, Conflict of interest, Corporations, Governance, Globalization

## Abstract

Efforts to adopt public health policies that would limit the consumption of unhealthy commodities, such as tobacco, alcohol and ultra-processed food products, are often undermined by private sector actors whose profits depend on the sales of such products. There is ample evidence showing that these corporations not only try to influence public health policy; they also shape research, practice and public opinion. Globalization, trade and investment agreements, and privatization, amongst other factors, have facilitated the growing influence of private sector actors on public health at both national and global levels. Protecting and promoting public health from the undue influence of private sector actors is thus an urgent task. With this backdrop in mind, we launched the “Governance, Ethics, and Conflicts of Interest in Public Health” Network (GECI-PH Network) in 2018. Our network seeks to share, collate, promote and foster knowledge on governance, ethical, and conflicts of interest that arise in the interactions between private sectors actors and those in public health, and within multi-stakeholder mechanisms where dividing lines between different actors are often blurred. We call for strong guidance to address and manage the influence of private sector actors on public health policy, research and practice, and for dialogue on this important topic. Our network recently reached 119 members. Membership is diverse in composition and expertise, location, and institutions. We invite colleagues with a common interest to join our network.

## Background

The consumption of unhealthy commodities, such as cigarettes, alcohol and ultra-processed food products is associated with ill-health [1]. Markets in high income countries are saturated with these products, and therefore private sector actors are rapidly shifting to penetrate emerging markets in low and middle income countries [1]. Efforts to develop public policies to limit consumption of these unhealthy commodities across the globe, including from governments, have been undermined by private sector actors [2] (or ‘industry actors’, two terms that we used interchangeably in the present manuscript), with the support of trade associations and front groups. Industry actors employ a diverse range of political strategies to influence public health research, policy, and practice, to persuade governments and the public of their potential legitimacy, and to present themselves as responsible partners in public health (further referred to as ‘undue influence from private sector actors’ in this commentary) [3]. This undue influence has been exposed during the current COVID-19 pandemic, with corporations profiting from the pandemic through, for example, their lobbying aggressively for classifying unhealthy commodities, such as cigarettes and alcohol, as essential goods, and by projecting themselves as responsible companies by donating personal protective equipment and medical devices to governments under national response to COVID-19 [4].

Kickbusch et al. refer to a broader range of “strategies and approaches used by the private sector to promote products and choices that are detrimental to health”, as the ‘commercial determinants of health’ [5]. Kickbusch et al. explain that marketing, lobbying and other undue influence from private sector actors are led by global drivers of ill-health [5]. Undue influence operates at national and global levels, where globalization, trade and investment agreements, and privatization, amongst other neo-liberal policies, have indeed facilitated the growing influence of private sector actors on public health. In turn, private sector actors also shape these global drivers of ill-health, through their participation in platforms like the World Trade Organization and Codex Alimentarius. The United Nations Sustainable Development Goal (UN SDG) 17 is perhaps the best illustration of the increasing ideological pressure to develop public private partnerships as a way of promoting human wellbeing. In the wake of continuing declines in public funding for research and advocacy, many individuals and institutions, including the World Health Organization (WHO), now engage with these private sector actors without any substantive conditions or constraints to avoid undue influence. Within multi-stakeholder mechanisms, dividing lines between different actors are often blurred.

In response, there is a growing counter-response by public health professionals concerned about undue influence from private sector actors, particularly the impact on governance, ethics, and conflicts of interest. These concerns have resulted in the publication of numerous peer-reviewed articles, casebooks, blog posts, reports, and research meetings that have highlighted these issues, and provided evidence of the lack of transparency and appropriate guidance to address and manage undue influence [6]. The WHO Framework Convention on Tobacco Control is the only international treaty that has been successful in protecting public policies from undue influence from private sector actors, through the implementation of its Article 5.3 on the engagement with the tobacco industry [7]. Similar approaches have yet to be adopted to protect public health from undue influence from private sector actors in the alcohol, gambling, and ultra-processed food industries, amongst others. Policies to address any undue influence are not only needed at the UN and government levels, but also for academia and civil society organizations.

## Main text

With this global backdrop in mind, a group of public health professionals from around the globe, concerned about the undue influence of private sector actors on public health were invited to a meeting in February 2018 at the Faculty of Health Sciences at the American University of Beirut, Lebanon, funded by the International Development Research Center (IDRC), Canada. There, we discussed our research experiences and knowledge; and we set a research agenda for the protection and promotion of public health policies and practices. The meeting started with the recognition that accepting funds from and engaging with certain private sector actors, such as those that produce and sell unhealthy commodities and their front offices, result in ethical and practical challenges. Towards the end of the meeting, we decided to launch the “Governance, Ethics, and Conflicts of Interest in Public Health” Network (GECI-PH Network). In doing so, we used the medical definition of conflict of interest (COI), also used by the WHO, where a set of conditions in which professional judgment concerning one interest may be unduly influenced by another interest, such as financial benefit [8]. We defined ethics in this context as value-based justification of policies and programs to advance population health, while governance for public health refers to the interactions of state institutions and non-state actors in collectively creating conditions for optimizing population health [9].

We chose a network as a working mechanism since networks connect researchers that are working in different global regions around a common research agenda, and allows the creation of joint projects while simultaneously allowing for support, mentorship and guidance on individual projects; and sharing of lessons learned in one context to inform another. Networks thus are spider webs of connections for maximum co-learning and co creation opportunities. They can be loose and strong simultaneously, building on assets, expertise, and relationships of each member.

The network has seven objectives: i) sharing knowledge of undue influence from private sector actors in public health research, practice, and policy; ii) documenting the governance, ethical, and COI issues that arise in the interactions between individuals and institutions in public health and those from the private sector; iii) encouraging research into and providing recommendations about best practices in governance and managing of ethical and COI concerns in these interactions; iv) sharing lessons learned and responses related to undue influence from private sector actors on public health at a local, national, regional and global level; v) actively demanding limits on the engagement with industry actors in public health; vi) fostering policy dialogue around this topic; and vii) conducting research that helps set priorities on governance, ethics, and conflicts of interest (COI) in public health.

After our first meeting, we undertook a Delphi process amongst our members and their extended network (only including those individuals who had no COI) to identify relevant research gaps and priorities. The Delphi questionnaire included research topics that were identified by participants of the 2018 GECI-PH meeting and from a recent research agenda on transnational corporations and health developed by Baum and Anaf [10]. Baum and Anaf’s research agenda focuses on the impact of private sector actors on health and equity; effectiveness of government efforts to address these negative impacts; the role and work of advocates; and regulation of capitalism for a healthier and more equitable private sector [10]. Principal research gaps identified through our Delphi process related to: i) identifying tactics used by corporations to unduly influence public health policy, research and practice; ii) identifying frameworks, policies, and tools that can be used to support public health to govern effectively and control COI and undue influence from private sector actors; iii) developing communication messages to increase awareness of the ethical and practical challenges associated with COI and undue influence from private sector actors; iv) exploring global trade barriers that impede an agenda of COI management for governments; v) identifying policies to govern and limit conflicts of interest in relation to universities and engagement with corporate actors.

We had a second annual meeting in Beirut in March 2019, attended by 25 members. During that meeting, we discussed the results of the Delphi process and collectively decided to focus on four research gaps, as a network: i) mapping tactics of health-harming industries to unduly influence public health policy, practice and research; ii) policies to limit and manage COI and undue influence from private sector actors in public health; iii) best strategies for strengthening understanding of and capacity to manage COI amongst public health actors; and iv) global trade policies that have enabled undue influence from private sector actors on public health. Further work funded through a Wellcome Trust grant will explore new methodologies for researching the relationship between corporations and public health.

The GECI-PH Network provides a platform for its members to interact, share relevant resources and coordinate research and advocacy efforts. The Network had expanded since its inception and recently reached 119 members. Membership is diverse in composition and expertise (experts on the alcohol, tobacco, food and other industries), location (all continents), and institutions (academia, practitioner, civil society). Our network is accessible through its website (https://aub.edu.lb/fhs/Pages/GECI.aspx) and Twitter handle (@GECI_ph), sends regular updates to its members by email and publishes a newsletter twice a year. We organized two panel discussions at the 2018 American Public Health Association and European Public Health Association annual conferences. We organized a World Leadership Dialogue at the 2020 World Congress on Public Health. With support from IDRC, we have funded three research projects led by early career researchers and/or researchers from low and middle income countries, on topics aligned with the objectives of our Network. We have supported members facing undue influence from private sector actors on their institutions by providing support for lobbying and advocacy to prevent such co-option. The Network has also organized a series of webinars on the commercial determinants of health and solutions to address harmful corporate practices, with recordings available on our website.

## Conclusions

The GECI-PH network calls for appropriate guidance to address and manage undue influence from private sector actors on public health policy, research and practice, and aims to foster dialogue on this important topic. We acknowledge the increased research and advocacy actions around these issues by various institutions and individuals. We understand that each may come from slightly different backgrounds or starting points. We are eager to partner with other entities as our success is only in working together. We need to also expand our network beyond health, as private sector actors also influence public policy in other fields covered by the UN SDGs, including education (SDG4), water and sanitation (SDG6), climate (SDG13), peace and justice (SDG16). We strongly believe that our strength will be in our numbers, in our combined expertise and commitments, and in producing and disseminating evidence for decision making.

You could join the GECI-PH Network by sending an email to: gecoi.ph@gmail.com

## Data Availability

Not applicable.
